# Preparatory Strength Benchmarks for “Inverted Cross on Rings” in Male Elite and Junior Artistic Gymnasts

**DOI:** 10.3390/sports13050146

**Published:** 2025-05-14

**Authors:** Christoph Schärer, Eddy Yusof, Claudio Capelli

**Affiliations:** 1 Department of Elite Sport, Swiss Federal Institute of Sport Magglingen (SFISM), 2532 Magglingen, Switzerland; 2 Movement and Sport Science, Department of Medicine, University of Fribourg, 1700 Fribourg, Switzerland

**Keywords:** strength prerequisites, training, injury prevention, physical resilience

## Abstract

In men’s gymnastics, to build strength for static strength elements on rings (e.g., the Inverted Cross on Rings: ICR), general and ring-specific conditioning exercises are used. We aimed to examine the differences between elite and junior athletes in ring-specific strength in the ICR and the one-repetition maximum (1RM) in two conditioning exercises (Inverted Cross with Dumbbells: ICD; Seated Overhead Barbell Press: SOBP), to analyze the relationship between strength in the ICR and ICD and SOBP, and to establish preparatory strength benchmarks for ICR. Nine elite (20.97 ± 1.91 years, 66.01 ± 5.03 kg) and ten junior athletes (16.72 ± 0.55 years, 61.10 ± 7.9 kg) performed a maximum strength test for the ICR (five-second hold with pulley) and a 1RM test for the SOBP and ICD. Elite athletes were significantly stronger in the ICR and SOBP (22.36% and 33.2%; *p* < 0.001) but not in ICD (*p* = 0.13). Strong correlations (r > 0.65, *p* < 0.01) suggest that these exercises support strength development for the ICR. Although, the transfer into ring-specific strength must be trained separately, the benchmarks (SOBP: 150% body weight; ICD: 66%) offer coaches guidelines for optimizing training, prevent injury and promote physical resilience of elite athletes.

## 1. Introduction

Artistic gymnastics has evolved significantly over the past few decades. The fundamental reform of the scoring system by the International Gymnastics Federation (FIG) in 2006, which replaced the maximum score of 10 with an open judging system, along with advancements in equipment (e.g., more elastic floor surfaces) and improved movement techniques, has led to a substantial increase in the level of difficulty across all apparatuses over the last 20 years [1]. However, this was accompanied by a decline in execution quality [2].

On rings, dynamic and static strength elements have become the most important elements in order to increase the difficulty level of the competition routine and to succeed at the international level [3]. Up to six strength elements can be presented in one routine. These elements are categorized in the CoP into *strength and hold* elements and *swing to strength hold* elements and each element is assigned a difficulty value from A (0.1-point bonus for the difficulty score) to H (0.8-point bonus). Athletes must perform at least one element from each element groups and the sum of the bonuses for the seven most difficult elements in the competition routine (+dismount), along with other bonuses (e.g., requirements for element groups), account for a large proportion of the difficulty score. It is obvious that the athletes are striving to perform the most difficult elements as cleanly as possible. In order to avoid deductions in the execution score (E-score), an element must be held for a minimum of two seconds without any deviation from the perfect hold position [4]. The most rewarding and most frequently performed elements are therefore those with a difficulty value of D and higher (bonus > 0.4 points). Within the CoP [4] 35 strength and hold elements and 16 swing to strength hold elements are classified as D or higher.

In the 2025 Code of Points (C.o.P.), [4] the difficulty value of the element “Inverted Cross on Rings” was increased from C (0.3-points bonus) to D (0.4-points bonus). Simultaneously, the difficulty value of all *swing-to-strength* elements and *strength* elements concluding in the Inverted Cross position was raised by 0.1 points. This change encourages coaches and athletes to invest in targeted strength and technique training for the “Inverted Cross on Rings” to successfully incorporate it into their competition routines in order to increase the difficulty value of the competition routine on rings.

**Figure 1:**
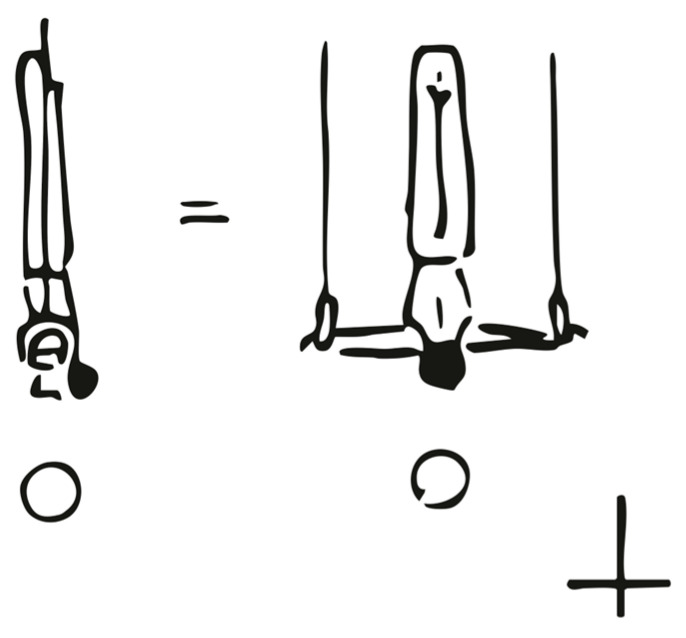
Inverted Cross on Rings: Depiction of the perfect holding position of the strength hold element “Inverted Cross on Rings” (FIG [4], p. 67).

For all static strength elements, the perfect position for the “Inverted Cross on Rings”, which must be held for two seconds in order to be recognized by the judges without deduction, is with arms in a perfectly horizontal position (Figure 1).

This suggests that the muscles of the upper body contract isometrically, meaning the muscle length remains nearly the same while the muscle tension changes [5]. Unlike classic isometric exercises, where the force is applied against an insurmountable resistance, gravity must be overcome constantly on rings while holding the element. Therefore, remaining in the static position on rings elicits at least partly an eccentric muscle contraction. In line with this, Schärer et al. [3] demonstrate the positive effects of eccentric exercises on the specific static strength on rings in their study.

In contrast to other static elements on rings (e.g., swallow, iron cross) where center of gravity is between or beyond the rings, the center of gravity while maintaining the “Inverted Cross on Rings” is above the rings. Therefore, this holding position is—from a biomechanical point of view—more unstable than strength elements with a horizontal body position. Accordingly, there is a higher risk of falling forward or backward compared to other static strength elements. This may explain why preparatory strength accounts for only up to 48% of maximum strength when holding this element on rings [3]. The technical level of execution of the element (e.g., balance skills in the holding position), plays an equally important role, which could make this element particularly interesting for junior athletes, who have yet to fully develop ring-specific maximum strength.

Nevertheless, preparatory strength plays an important role in protecting the athletes’ joints, tendons and muscles from injury. Therefore, it is essential to systematically develop the necessary preparatory strength for static elements on the rings already at the junior level. For this reason, it is also important to assess the maximal strength levels of junior athletes and to regularly monitor training progress. This allows a continuous adjustment and optimization of the training process, as well as the elimination of inefficient training methods. Ultimately, these measures contribute to building athletes’ physical robustness and to preventing overuse injuries.

In gymnastics training, traditionally, concentric barbell and dumbbell exercises that train similar muscle groups in similar arm positions have been used by practitioners to improve the preparatory strength [3]. However, not all traditional preparatory strength exercises are highly correlated with the target element on the rings [6]. To maximize the efficiency of strength training, it is important to understand the relationship between conditioning exercises and maximum strength in the target element and to know the required level of preparatory strength in each training exercise. Training efficiency is particularly important in highly training-intensive sports such as artistic gymnastics, in order to avoid placing unnecessary additional strain on athletes alongside the already high load from technical training.

Therefore, this study primarily aimed to provide insight into the differences in ring-specific and preparatory strength levels between elite and junior athletes and, secondly, to examine the relationship between maximal strength performing the element “Inverted Cross on Rings” (five-second holding time) and maximal strength (one-repetition maximum) in two traditional, sport-specific strengthening exercises using a barbell and dumbbells. Third, we aimed to calculate strength benchmarks in preparatory strength for the element “Inverted Cross on Rings”.

We hypothesized that elite athletes would demonstrate significantly greater strength in all tested specific and preparatory exercises and that the 1RM of preparatory strength exercises was significantly correlated with maximum strength in “Inverted Cross on Rings” for elite and junior athletes.

## 2. Materials and Methods

Nineteen male gymnasts, including nine elite athletes (age: 20.97 ± 1.91 years, height: 169.94 ± 5.4 cm, weight: 66.01 ± 5.03 kg) and ten junior athletes (age: 16.72 ± 0.55 years, height: 171 ± 7.42 cm, weight: 61.10 ± 7.9 kg), volunteered to participate in the study. Inclusion criteria required that athletes were members of the national junior or elite gymnastics team and participated in professional training (>25 h/week) at the national and/or a regional training center.

All included athletes were informed about the test procedures. Informed consent was obtained from all subjects involved in the study. The study was conducted in accordance with the Declaration of Helsinki, and approved by the Ethics Committee of Bern (project-ID: 2018-00742, 7 June 2018). According to a prior oral interview, all athletes were healthy and able to perform the maximum strength tests without restrictions.

Each athlete had to perform three strength tests during a training session. First, maximum strength was determined for the element “Inverted Cross on Rings” using a pulley system. Second, the preparatory strength was determined with two one-repetition-maximum (1RM) tests with dumbbells (Inverted Cross with Dumbbells) or a barbell (Seated Overhead Barbell Press). After an individual gymnastics-specific warm-up lasting 20 min, the three tests were performed each separated by at least 15 min recovery. The preparatory strength tests were performed in a self-chosen order.

### 2.1. Maximum Strength Test of “Inverted Cross on Rings”

Maximum strength was determined for the “Inverted Cross on Rings” element (Figure 1) using a pulley system with counterweight to facilitate the exercise. The holding position had to be achieved by lowering from Handstand into “Inverted Cross on Rings”. Maximum strength was defined as the maximum resistance that could be held for five seconds (bodyweight—counterweight). The counterweight was selected to match the individual’s strength level allowing each to hold the static position accurately for a maximum of five seconds. In the first attempt, the athletes had to hold 50% of their body weight (rounded to 2.5 kg) for 3 s. Where the load was perceived as light or very light, the counterweight for the second attempt, which was held for 5 s, was reduced by 2.5 or 5 kg, respectively. Further attempts were made according to this method until the athlete was able to hold the position for precisely 5 s. Between two attempts, athletes had three to five minutes recovery. Preliminary trials during training two days before the tests helped the athletes to define their approximate counterweight.

**Figure 2:**
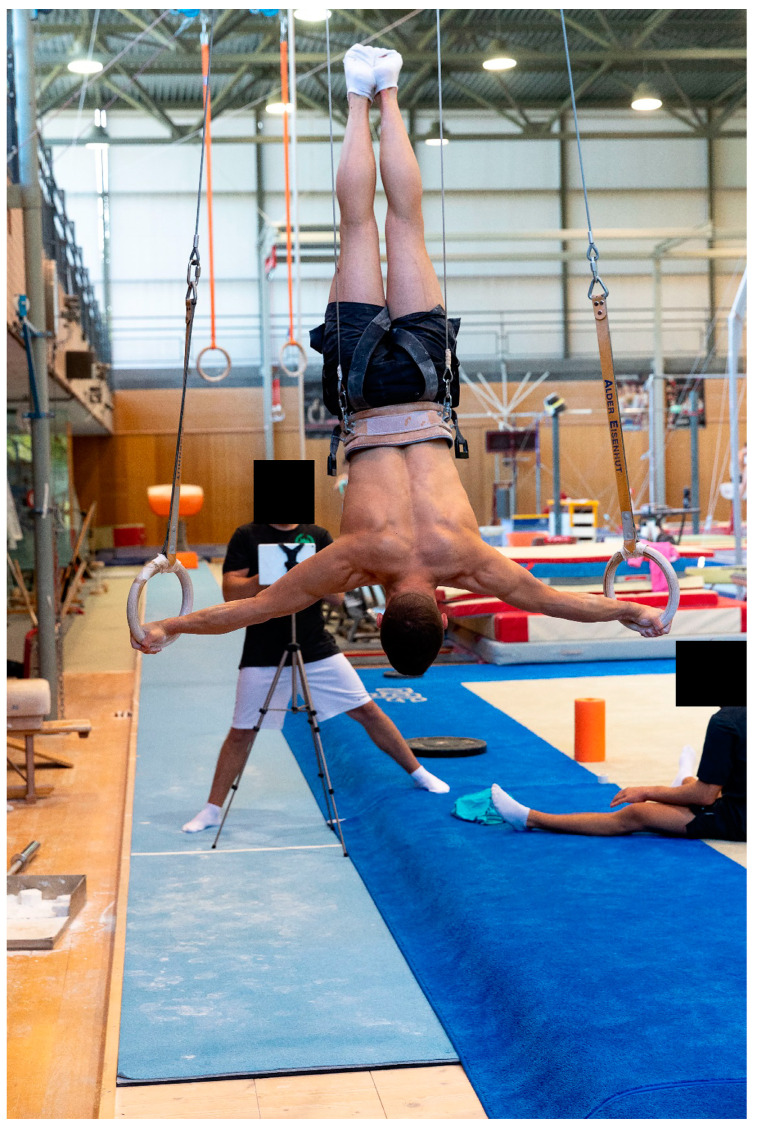
Maximum strength test of “Inverted Cross on Rings”. Maximum strength test of the element “Inverted Cross on Rings” with the counterweight device to facilitate maintaining the holding position for five seconds.

Trials were recorded using an iPad (iPad Pro 9.7”, Apple Corporation, Cupertino, CA, USA) placed at a distance of three meters in the frontal plane and at the height of the rings (1.3 m). Holding times and holding position (shoulder angle) were verified using Dartfish video analysis software (Dartfish SA, Fribourg, Switzerland). Time measurement started when the athlete reached a stable hold position for at least two subsequent video frames and stopped when the athlete departed from the holding position. Attempts were only valid if the maximum angular deviation for the recognition of the element specified in the C.o.P. did not exceed 45° (FIG, 2022) [4] (Figure 2).

### 2.2. Tests with 1RM of Specific Strengthening Exercises

The traditional conditioning exercises “Seated Overhead Barbell Press” and “Inverted Cross with Dumbbells” were used to determine the preparatory strength need for the “Inverted Cross on Rings”.

#### 2.2.1. Seated Overhead Barbell Press

**Figure 3:**
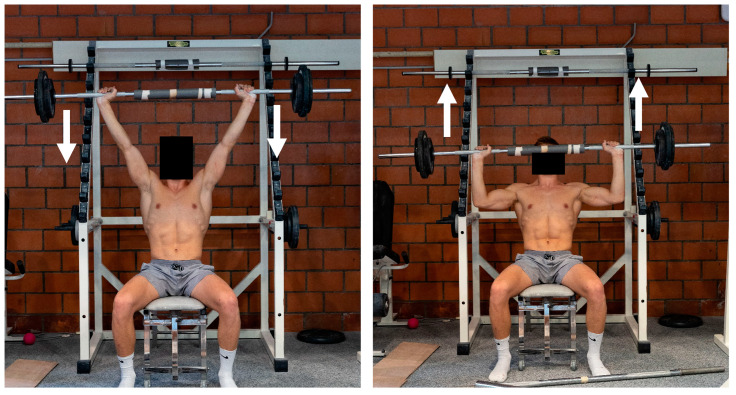
Test with 1RM of “Seated Overhead Barbell Press”. (**left**): starting and end position; (**right**): lowest (reverse) position.

To assess the 1RM of the “Seated Overhead Barbell Press” exercise, the athlete was seated on a bench with a backrest fixed at a 70° angle, which was placed inside a barbell rack. The bar was lifted from the rack with the help of a coach to the starting position (arms straight up). The bar was then lowered to a position where the elbows were at a 90° angle and pushed back to the starting position. The barbell grip width was set so that in the bottom position the upper arms were horizontal and the forearms were vertical (Figure 3, right). In the initial trial, the movement was executed three times with a load equivalent to 50% of the athlete’s body weight (rounded to 2.5 kg). If the load was perceived as light or very light, the weight was increased by 5, 10 or 20 kg in the following one-repetition attempt. For the following attempts, the weight was adjusted individually. In this way, all athletes reached their 1RM in a maximum of five attempts, the maximum number of attempts used in other studies [7]. Athletes had a five-minute break between attempts in order to recover completely.

#### 2.2.2. “Inverted Cross with Dumbbells”

**Figure 4:**
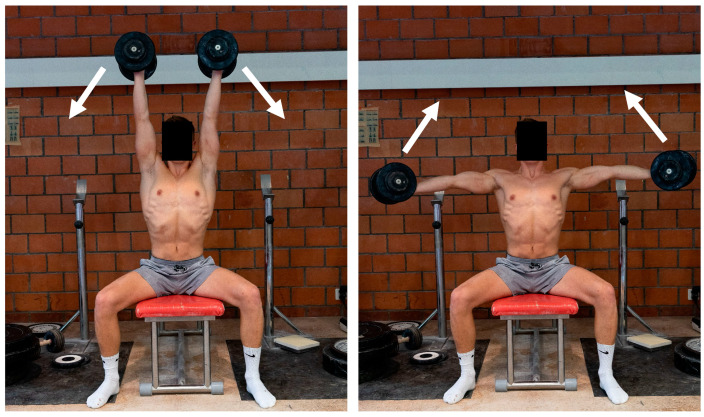
A 1RM test of the preparatory strength exercise “Inverted Cross with Dumbbells” (**left**): starting and end position; (**right**): lowest (reverse) position).

The 1RM of “Inverted Cross with Dumbbells” exercise was determined seated on a bench. With the help of a coach, the dumbbells were raised to the starting position (arms abducted to a vertical position). The fully extended arms were then lowered laterally to a horizontal position before returning to the starting position. During execution, the elbows remained extended and the upper body was allowed to lean backwards by a maximum of 20° during the concentric movement phase (Figure 4). In terms of repetitions and rest periods, this test was identical to the “Seated Overhead Barbell Press”. For the initial attempt, the weight of the dumbbells corresponded to 15% of the body weight. For the subsequent attempts, the load was increased by 2 kg, respectively 4 kg. The dumbbell weight in the further attempts was increased individually.

### 2.3. Statistical Analyses

Maximum strength values of the three tests were calculated as a percentage of the athlete’s body weight. Descriptive statistics were run and normal distribution was confirmed using Shapiro–Wilk test for all data and separately for elite and junior athletes.

T-tests and effect sizes (Cohen’s d) were calculated to determine significant differences between elite and junior athletes. To assess the general relevance of preparatory strength for maximum strength in the “Inverted Cross on Rings”, a multiple linear regression analysis was performed. While the strength exercises examined may appear similar in terms of the general physical capacities they engage, they differ in key biomechanical and functional aspects—such as joint angles, muscle activation patterns, and movement dynamics. Therefore, Pearson correlations (r) and explained variance (R^2^) between these strength measures were calculated, to examine the relevance of each of the two preparatory strength training exercises for maximum strength in the “Inverted Cross on Rings. Finally, 1RM benchmarks of the preparatory strength exercises were calculated using the linear function (y = ax + b) to determine the level of strength need to perform the “Inverted Cross on Rings” without a counterweight. The significance level was set to *p* < 0.05.

The achieved statistical power was calculated to be 94% (*t*-test) and 98% (Pearson correlation), respectively, based on an alpha level of *p* > 0.05, the current group sizes and a large effect size (Cohen’s d = 1.5) or strong correlations (r = 0.7).

## 3. Results

All participating athletes were able to complete the three strength tests.

### 3.1. Maximum Strength Level of Junior and Elite Athletes

The comparison between junior and elite athletes (Table 1) revealed significantly higher strength among elite athletes than juniors, with strong effect sizes for the “Inverted Cross on Rings” (+33.17%; d = 2.41) and “Seated Overhead Barbell Press” (+22.36%; d = 1.81). In contrast, no significant difference was observed between the two groups for the “Inverted Cross with Dumbbells” (elite > juniors: +11.07%; *p* = 0.13; d = 0.74). Individual results of all athletes can be found in the Supplementary Material (Table S1).

### 3.2. Relationship Between the “Inverted Cross on Rings” and the 1RM of “Seated Overhead Barbell Press” and “Inverted Cross with Dumbbells”

A multiple linear regression analysis was conducted to predict maximal strength performance in the “Inverted Cross on Rings” based on two preparatory strength exercises: (1) “Inverted Cross with Dumbbells” and (2) “Seated Overhead Barbell Press”. The model was statistically significant, F(2, 16) = 11.79, *p* < 0.001, and explained 60% of the variance in maximum strength of “Inverted Cross on Rings” (R^2^ = 0.60, Adjusted R^2^ = 0.545, RMSE = 8.74).

Considering the relevance for ring-specific maximum strength of the two different preparatory strength exercises, in the elite group, significant relationships were observed between the “Inverted Cross on Rings” and both conditioning exercises. In contrast, among junior athletes, only “Inverted Cross with Dumbbells” was significantly correlated with the maximum strength performing the element on rings while no significant relationship was found for the “Seated Overhead Barbell Press” (*p* = 0.57). For the entire athlete cohort, 1RM from “Inverted Cross with Dumbbells” explained 42% (R^2^) and “Seated Overhead Barbell Press” explained 53% (R^2^) of maximum strength in the “Inverted Cross on Rings”, respectively (Table 2).

### 3.3. Preparatory Strength Benchmarks for the “Inverted Cross on Rings”

**Figure 5:**
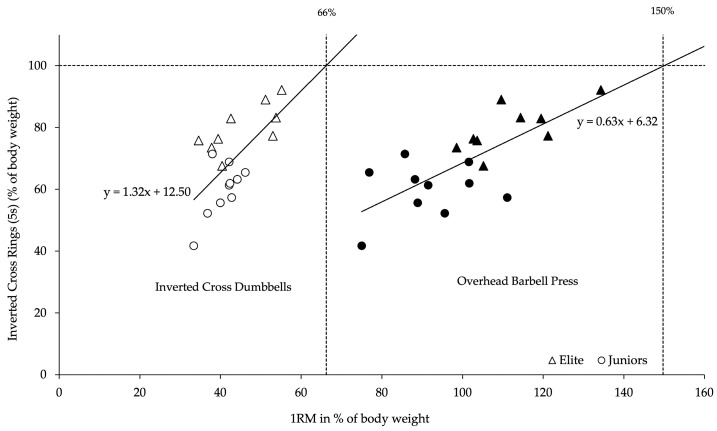
Preparatory strength benchmarks as a percentage of body weight: Linear regression analysis between maximum strength in the “Inverted Cross on Rings” and the 1RM of the conditioning exercises “Inverted Cross with Dumbbells” (**left**) and “Seated Overhead Barbell Press” (**right**) (Elite athletes: n = 9; Junior Athletes: n = 10).

The linear regression of preparatory strength revealed that the required 1RM in conditioning exercises to maintain the “Inverted Cross on Rings” was 150% of body weight for the “Seated Overhead Barbell Press” and 66% of body weight for the “Inverted Cross with Dumbbells”, respectively (Figure 5).

## 4. Discussion

This study analyzed the differences in maximal strength levels between elite and junior athletes performing the “Inverted Cross on Rings” (5 s holding time) and two preparatory strength training exercises (1RM) using dumbbells or a barbell. Furthermore, the relationships between maximal strength in the “Inverted Cross on Rings” and the conditioning 1RM tests were examined, and strength benchmarks for the 1RM tests were determined to assess the required strength for holding the Inverted Cross on Rings without external support.

### 4.1. Maximum Strength Level of Junior and Elite Athletes

Elite athletes demonstrated significantly greater maximal strength than junior athletes in two of the three strength tests. In the “Inverted Cross on Rings” and “Seated Overhead Barbell Press”, elite athletes were up to 33% stronger relative to their body weight compared to junior athletes. However, no significant strength difference between groups was observed in the conditioning exercise “Inverted Cross with Dumbbells”, although elite athletes achieved a mean 1RM that was 10% higher than that of junior athletes.

The results confirm that elite athletes demonstrate superior maximal strength levels. This advantage is likely attributable to their advanced stage of development (four years older than junior athletes), greater experience in strength training, and their more extensive and structured professional training. It is also noteworthy that, despite their higher body weight (+5 kg), elite athletes achieved higher relative maximal strength values in all strength tests compared to junior athletes. This suggests that elite athletes possess greater muscle mass than junior athletes and that a certain amount of muscle mass is necessary to maintain strength-hold elements on the rings.

However, the findings also highlight the importance of regularly assessing maximal strength levels—both in ring-specific elements and preparatory strength training exercises. These assessments provide essential insights for adjusting training strategies and to enhance the effectiveness of training interventions. However, regular maximal strength monitoring is only meaningful if the selected test exercises effectively differentiate strength levels. This was (given the small differences observed between elite and junior athletes) not the case in this study for the “Inverted Cross with Dumbbells”. Due to the large lever arm created by fully straight arms, a 1 kg increment in dumbbell weight likely represents a too large step to detect subtle strength differences accurately. To improve measurement precision, either smaller weight increments should be available for dumbbells, or a force measurement device (e.g., a motor-driven cable pulley system with an integrated force measurement function) should be used to assess maximal preparatory strength more accurately [3]. A smaller weight increment is also important to precisely achieve the desired stimulus in strength training.

### 4.2. Relationship Between the “Inverted Cross on Rings” and the 1RM of “Seated Overhead Barbell Press” and “Inverted Cross with Dumbbells”

The results of the multiple linear regression analysis suggest that both preparatory strength exercises contribute to the maximum strength of the “Inverted Cross on Rings” (R^2^ = 0.60; *p* < 0.01). However, it is important to distinguish between general preparatory strength exercises performed with bent arms and those performed with straight arms. In this context we found strong and significant correlations between maximum strength in “Inverted Cross on Rings” and 1RM in both preparatory strength exercises when considering the combined results of elite and junior athletes. Maximal strength in the “Inverted Cross on Rings” was explained by 42% (Inverted Cross with Dumbbells) or 53% (Seated Overhead Barbell Press) by the 1RM of the conditioning exercises, respectively. These findings are consistent with previous studies that examined the relationship between the 1RM of concentric preparatory strength exercises and maximal strength in static strength elements on rings [6]. It can therefore be assumed that preparatory strength is equally important for the “Inverted Cross on Rings” as it is for the Swallow, Support Scale, or Iron Cross. This is contrary to expectations, as the “Inverted Cross on Rings” is biomechanically more unstable than the other strength-hold elements due to the body’s center of mass being positioned above the rings. Apparently, the transfer of preparatory strength appears to be similarly direct at a certain technical level as it is for other strength-hold elements on rings (e.g., Swallow, Support Scale or Iron Cross).

However, it is important to distinguish between general preparatory strength exercises performed with bent arms and those performed with straight arms. Exercises performed with bent arms offer the advantage of allowing higher loads to be moved, thereby promoting long-term increases in general maximal strength of the involved muscle groups what could have a preventive effect against injuries [8]. Additionally, these exercises place less leverage stress on the shoulder joints, making them generally more tolerable for athletes. It is particularly important that, especially for young athletes, a general maximal strength foundation in the upper extremities should first be established. This not only helps to develop muscular potential that may be transferred later into specific ring strength, but also fosters a certain robustness, allowing athletes to train for longer periods while remaining free of injury. In contrast, preparatory strength exercises with straight arms are often more specific to the holding position on rings, as they directly engage the muscles required for performing static strength elements on rings [9,10,11,12].

Examining the correlations for elite and junior athletes separately, it is noticeable that the 1RM of the “Seated Overhead Barbell Press” shows no significant correlation with the maximal strength in the “Inverted Cross on Rings” among junior athletes. It demonstrates that both specific and non-specific preparatory strength exercises need to be trained separately. In our case, it suggests that the junior athletes have likely only engaged in specific strength training up until now. Specific training for the target element improves technique and enhances the muscular efficiency required to maintain the hold position, thereby facilitating a more effective transfer of preparatory strength to the target element [13]. This is even more important when considering that in competition routines, the strength-hold elements are often performed from a swing element. Merz et al. [14] have shown that high forces (>body weight) act during swing to strength elements because the swinging body must be decelerated into the hold position. Furthermore, a high rate of force development plays a crucial role in being able to maintain the swing to strength element, and this may be facilitated by a highly efficient work pattern of the involved musculature.

In any case, effective training requires a combination of all forms of strength training: general muscle preparation through bent-arm exercises, specific muscle preparation through straight-arm exercises, and direct practice of the static hold on the rings. Only through this comprehensive approach can the preparatory strength gains be effectively transferred to the hold position of the static element on rings. In order to increase the effectiveness of strength training for the “Inverted Cross on Rings”, technical aspects such as balance and multidirectional force application should also be trained in combination with the two strength exercises mentioned. The dynamic eccentric-concentric strength exercise “Handstand—Inverted Cross—Handstand” on rings executed with the pulley system may be suitable to simulate these aspects.

Notably, this study found that the disparity between junior and elite athletes was greater in the “Seated Overhead Barbell Press” exercise compared to the other two maximum strength exercises and “Seated Overhead Barbell Press” was not significantly correlated with “Inverted Cross on Rings” for junior athletes (R^2^ = 4%). In general, junior athletes are often not accustomed to lifting maximal loads. Additionally, it can be assumed that they were not familiar with this exercise at all, which may explain this result.

### 4.3. Preparatory Strength Benchmarks for the “Inverted Cross on Rings”

The estimated minimal preparatory strength required to hold the “Inverted Cross on Rings” was 150% of the body weight for the conditioning exercise “Seated Overhead Barbell Press” and 66% for the “Inverted Cross with Dumbbells”. These values are similar to previous studies that investigated preparatory strength benchmarks of conditioning exercises with extended and bent arms (swallow in supine position with dumbbells and bench press) for other static strength elements on rings (e.g., Iron Cross, Swallow, Support Scale) [3,6].

Preparatory strength benchmarks may serve as valuable reference points for coaches and athletes throughout the training process. First, they facilitate the classification of the individual preparatory strength level and provide a means to track its development following intensive strength training periods. Second, these benchmarks provide guidance on whether training should prioritize further increases in preparatory strength or focus on transferring this strength to specific performance demands on the rings.

From a general scientific perspective, the number of athletes who participated in this study may be considered relatively small. However, in relation to the total number of professional athletes in this discipline, the sample size can be regarded as remarkably substantial. Accordingly, the results of this study may be considered representative of the corresponding population, particularly given the high-performance level of the participating athletes.

For future research, it would be important to validate the findings under practical conditions with a greater number of athletes and, especially with athletes who are already able to hold the “Inverted Cross on Rings” without external support. Only then, the results can be considered generalizable.

## 5. Conclusions

This study demonstrated that the specific and preparatory strength of elite athletes for the element “Inverted Cross on Rings” is significantly greater than that of junior athletes, despite their higher body weight.

Furthermore, it could be shown that maximal strength (1RM) in two commonly used preparatory strength training exercises “Seated Overhead Barbell Press” and “Inverted Cross with Dumbbells” show strong significant correlations with maximal strength in the static strength element “Inverted Cross on Rings” (except “Seated Overhead Barbell Press” for juniors). Consequently, it can be assumed that these exercises may be beneficial for strengthening the musculature involved in the “Inverted Cross on Rings” and for preparing the passive and active structures of the shoulder girdle for the loads experienced on the rings. However, it must be noted that the intensity of both preparatory exercises cannot be precisely controlled in the same manner. For “Inverted Cross with Dumbbells”, small weight increments are necessary due to the extended arms and the long lever arm. Furthermore, technique training must be used to improve efficiency in the hold position what enables a more direct transfer of preparatory strength to the strength-hold element on rings. However, it is important to note that maximal strength exercises performed with bent arms primarily enhance general strength capacities. These exercises exert lower lever forces on the shoulders and can therefore be safely implemented even at the junior level.

The calculated preparatory strength benchmarks for the two conditioning exercises in this study provide coaches with valuable reference value for the necessary levels of conditioning maximal strength, thereby facilitating a more structured training process. An individually optimized, highly structured strength training program, beginning at the junior level, prepares athletes for the high physical demands of elite sport and contributes to their robustness. Such training can help prevent overload-related injuries and foster a certain degree of physical resilience in athletes.

## Figures and Tables

**Table 1:** Mean values ± standard deviation of the maximum strength test of “Inverted Cross on Rings” (5 s holding time, body weight—minimal counterweight) and the one-repetition maximum (1RM) of the preparatory strength exercises “Inverted Cross Dumbbells” and “Seated Overhead Barbell Press” in percent of body weight (% of BW). ***: Significant differences between elite and junior Athletes (*p* < 0.001).

	Inverted Cross on Rings (% of BW)	Inverted Cross Dumbbells (1RM in % of BW)	Seated Overhead Barbell Press (1 RM % of BW)
All	69.29 ± 12.96	42.96 ± 3.18	101.33 ± 15.22
Elite	79.74 ± 7.75	45.34 ± 3.96	112.11 ± 11.38
Juniors	59.88 ± 8.66 ***	40.82 ± 1.90	91.62 ± 11.27 ***

**Table 2:** Pearson’s correlations (r) between the maximum strength performing the “Inverted Cross on Rings” and the preparatory one-repetition maximum (1RM) for the exercises “Inverted Cross Dumbbells” and “Seated Overhead Barbell Press” (significant correlations: *: *p* < 0.05; **: *p* < 0.01; ***: *p* < 0.001).

		Inverted Cross Dumbbells (1RM)	Seated Overhead Barbell Press (1RM)
Inverted Cross Rings (5s)	All	0.65 **	0.73 ***
	Elite	0.71 *	0.70 *
	Juniors	0.64 *	0.21

## Data Availability

The raw data can be found in the Supplementary Materials.

## Supplementary Materials

The following supporting information can be downloaded at: https://www.mdpi.com/article/10.3390/sports13050146/s1, Table S1: Raw data of the maximum strength test of “Inverted Cross on Rings”.

## Abbreviations

The following abbreviations are used in this manuscript:

ICR	Inverted Cross on Rings
ICD	Inverted Cross with Dumbbells
SOBP	Seated Overhead Barbell Press
1RM	One repetition maximum
