# Supplementary material for: Preparatory Strength Benchmarks for “Inverted Cross on Rings” in Male Elite and Junior Artistic Gymnasts
Source: Sports (Basel). 2025 May 14;13(5):146. doi: 10.3390/sports13050146 (PMC12115426; doi:10.3390/sports13050146)
Supplement: Supplementary file 1 [file sports-13-00146-s001.zip › sports-3580729-supplementary.pdf]

Supplementary Material:

Table S1: Raw data of the maximum strength test of «Inverted Cross on Rings» (5s holding time, body weight - minimal counterweight) and the one-repetition maximum (1RM) of the preparatory strength exercises «Inverted Cross Dumbbells» and «Seated Overhead Barbell Press» in percent of body weight (% of BW) of each elite and junior athlete.

**Athlete**	**Group**	**Age (y)**	**Height (cm)**	**Bodyweight (kg)**	**Inverted Cross on Rings (% of BW)**	**Inverted Cross Dumbbells (1RM in % of BW)**	**Seated Overhead Barbell Press (1 RM % of BW)**
1	Elite	24.8	163.0	63.3	92.1	55.2	134.3
2	Elite	22.8	174.0	74.3	83.2	53.8	114.4
3	Elite	21.8	164.5	58.6	82.9	42.6	119.5
4	Elite	19.1	169.0	63.3	76.3	39.4	102.7
5	Elite	20.9	166.0	66.0	77.3	53.0	121.2
6	Elite	19.9	166.0	68.4	89.0	51.2	109.6
7	Elite	20.3	178.0	72.4	75.8	34.6	103.6
8	Elite	20.4	175.0	66.0	73.5	37.8	98.5
9	Elite	18.8	174.0	61.8	67.6	40.4	105.2
10	Junior	16.4	159.0	52.5	71.4	38.0	85.7
11	Junior	16.4	159.0	45.0	55.6	40.0	88.9
12	Junior	16.8	177.0	64.0	68.8	42.2	101.6
13	Junior	16.9	180.0	68.1	63.2	44.2	88.2
14	Junior	17.2	174.0	64.9	65.4	46.2	76.9
15	Junior	17.8	175.0	68.3	52.2	36.8	95.6
16	Junior	16.4	177.0	70.7	61.3	42.2	91.5
17	Junior	15.8	170.0	58.5	57.3	42.8	111.1
18	Junior	17.0	173.0	60.3	41.7	33.4	75.0
19	Junior	16.6	166.0	58.7	61.9	42.4	101.7
